# Comparison of COVID-19 hospitalization costs across care pathways: a patient-level time-driven activity-based costing analysis in a Brazilian hospital

**DOI:** 10.1186/s12913-023-09049-8

**Published:** 2023-02-24

**Authors:** Ricardo Bertoglio Cardoso, Miriam Allein Zago Marcolino, Milena Soriano Marcolino, Camila Felix Fortis, Leila Beltrami Moreira, Ana Paula Coutinho, Nadine Oliveira Clausell, Junaid Nabi, Robert S. Kaplan, Ana Paula Beck da Silva Etges, Carisi Anne Polanczyk

**Affiliations:** 1 National Institute of Science and Technology for Health Technology Assessment (IATS) (project: 465518/2014-1), Federal University of Rio Grande do Sul (UFRGS), Porto Alegre, Brazil; 2 Graduate Program in Epidemiology, Federal University of Rio Grande do Sul (UFRGS), Porto Alegre, Brazil; 3 Internal Medicine Division, Federal University of Minas Gerais (UFMG), Belo Horizonte, Brazil; 4 School of Medicine, Federal University of Rio Grande do Sul (UFRGS), Porto Alegre, Brazil; 5 Hospital de Clínicas de Porto Alegre (HCPA), Porto Alegre, Brazil; 6 Harvard Business School, Boston, MA USA; 7 School of Technology, Pontifícia Universidade Católica do Rio Grande do Sul (PUCRS), Porto Alegre, Brazil

**Keywords:** Cost and cost analysis, Health care costs, Microcosting, Time-driven activity-based costing, TDABC, COVID-19

## Abstract

**Background:**

The COVID-19 pandemic raised awareness of the need to better understand where and how patient-level costs are incurred in health care organizations, as health managers and other decision-makers need to plan and quickly adapt to the increasing demand for health care services to meet patients’ care needs. Time-driven activity-based costing offers a better understanding of the drivers of cost throughout the care pathway, providing information that can guide decisions on process improvement and resource optimization. This study aims to estimate COVID-19 patient-level hospital costs and to evaluate cost variability considering the in-hospital care pathways of COVID-19 management and the patient clinical classification.

**Methods:**

This is a prospective cohort study that applied time-driven activity-based costing (TDABC) in a Brazilian reference center for COVID-19. Patients hospitalized during the first wave of the disease were selected for their data to be analyzed to estimate in-hospital costs. The cost information was calculated at the patient level and stratified by hospital care pathway and Ordinal Scale for Clinical Improvement (OSCI) category. Multivariable analyses were applied to identify predictors of cost variability in the care pathways that were evaluated.

**Results:**

A total of 208 patients were included in the study. Patients followed five different care pathways, of which Emergency + Ward was the most followed (*n* = 118, 57%). Pathways which included the intensive care unit presented a statistically significant influence on costs per patient (*p* <  0.001) when compared to Emergency + Ward. The median cost per patient was I$2879 (IQR 1215; 8140) and mean cost per patient was I$6818 (SD 9043). The most expensive care pathway was the ICU only, registering a median cost per patient of I$13,519 (IQR 5637; 23,373) and mean cost per patient of I$17,709 (SD 16,020). All care pathways that included the ICU unit registered a higher cost per patient.

**Conclusions:**

This is one of the first microcosting study for COVID-19 that applied the TDABC methodology and demonstrated how patient-level costs vary as a function of the care pathways followed by patients. These findings can be used to develop value reimbursement strategies that will inform sustainable health policies in middle-income countries such as Brazil.

**Supplementary Information:**

The online version contains supplementary material available at 10.1186/s12913-023-09049-8.

## Background

COVID-19 was detrimental to the financial well-being of healthcare systems. Hospitals used varying strategies to increase their capacity to care for the influx of COVID-19 patients [1]. A study of COVID-19 related care at 10 hospitals in Brazil showed high variability in inpatient care management and resource needs [2]. Having valid patient-level costs for COVID-19 care is necessary to inform and evaluate hospitals’ resource allocations and care delivery decisions.

Little evidence currently exists about the cost of inpatient care for COVID-19 patients. Time-driven activity-based costing (TDABC) is a microcosting technique applied to generate accurate patient-level cost information within the episode of care by estimating two factors: the capacity cost rate (CCR) of a resource and the period in which the resource is used [3–6]. TDABC offers a better understanding of the drivers of cost throughout the care pathway, providing information that can guide decisions on process improvement and resource optimization [7–10]. The literature recommends the use of TDABC not only because of its capability to drive cost evaluations but also because it can be a strong tool with which to identify inefficiencies and opportunities to improve patients’ flow of care and resource utilization [6, 11].

Brazil provides public health coverage (Sistema Único de Saúde – SUS) ensuring universal access to health. SUS is financed through tax collection without any patient co-payment or flat-rate and payments to hospitals are based on a government reference table [12], which lists all procedures/treatments reimbursement values. With the increasing use of contracts which include global budgets for public health care providers, the reimbursement table does not necessarily reflect either SUS’ expenditure or provider’s costs [13]. In this context, micro-costing studies in the perspective of public health reference centers are probably the best estimative of the real cost of a technology for the Public Health System [13].

The COVID-19 pandemic raised awareness of the need to better understand where and how patient-level costs are incurred in health care organizations, as health managers and other decision-makers need to plan and quickly adapt to the increasing demand for health care services to meet patients’ care needs [2, 14]. Although reference hospitals for COVID-19 treatment were able to take sufficient measures to ensure bed availability in the ICU and general infirmary during the first wave of the pandemic, they were not able to prevent health services to be overwhelmed during the second wave [15].

The aim of this study was to apply TDABC and estimate patient-level costs for the care of COVID-19 patients in Brazil. Additionally, to better understand patient care and resource needs according to the clinical conditions, this study aimed to evaluate cost variability between the in-hospital care pathways followed by different types of COVID-19 patients and between the patients’ clinical classification. To the best of our knowledge, this is one of the first microcosting study for the COVID-19 pandemic that used the TDABC, which, in the context of a worldwide scarcity of resources, warrants attention.

## Materials and methods

This was a prospective cohort study that applied the TDABC microcosting technique to estimate the cost per COVID-19 patient in a tertiary referral hospital.

### Study setting and patient sample

This study took place in a public academic hospital (Hospital A) that was a referral center for the treatment of patients with COVID-19 in Porto Alegre, State of Rio Grande do Sul, Brazil, as described by APBS Etges et al. [2]. Hospital A has 831 beds, including 41 emergency department beds and 150 intensive care unit (ICU) beds. The hospital dedicated 82 inpatient care beds and 105 ICU beds for the care of COVID-19 patients during the pandemic’s first wave. A convenience sample was selected consecutively from patients who were admitted at the hospital for COVID-19 treatment and discharged from March 2020 to August 2020. COVID-19 status was confirmed by reverse transcription-polymerase chain reaction (RT–PCR) at arrival.

### Patients’ clinical classification

The Ordinal Scale for Clinical Improvement (OSCI) was applied to classify patients’ clinical status [16]. The information evaluated by the scale included therapeutic strategies required for the patient’s treatment, such as high-flow oxygen, mechanical ventilation, and extracorporeal membrane oxygenation (ECMO). In addition to the OSCI score, the information included patients’ medical history, hospital length of stay (LoS), and mortality rate. All information was extracted from the hospital database containing the electronic medical records (EMRs).

### Cost measurements

Patient care costs were evaluated from a public teaching hospital perspective using the 8-step TDABC method [7], which was applied by a multidisciplinary team composed of physicians, health care professionals, and cost engineers (Table 1).

**Table 1:** Steps description of the TDABC method

Step	Activity	Description
1 and 2	Identify the study questions and build the care process map	Care activities and procedures were identified as well as all hospital units along patient’s care pathway (five pathways identified).^a^
3	Identify the main resources used in each activity and department	Resources were identified as:
		- Infrastructure of hospital units (emergency, regular ward, and intensive care); - Other resources: personnel (labor costs), medication, and exams.
4	Estimate the total cost of each resource group and department (time dependent)	- Labor costs: salaries per professional category plus related taxes; - Infrastructure costs: hospital direct fixed costs assigned for each hospital unit.^b^
5	Estimate the capacity of each resource and calculate the capacity cost rate (CCR-$/h)	- Labor: total salary divided by the contracted hours of each professional category;^c^ - Infrastructure (each hospital unit): total monthly costs divided by the number of hours the unit is open per month, then divided by the number of beds.
6	Analyze the time estimates for each resource used in an activity	- Health professionals visits’ LoT: sum of time required to complete all assigned tasks related to each patient care, calculated as the multiplication of:
		- Average LoT spent per care visit to patients (self-reported via online survey); - Number of visits to each patient (collected manually from patient’s EMRs);
		- Patient’s LoS: sum of time spent by patients at each hospital unit (collected manually from patient’s EMRs).
7	Calculate the overall cost of patient care	- Exam and medication: amount consumed per patient multiplied by the respective price per item;^d^ - Time related resources: CCR of each resource multiplied by the time consumed by each patient and summed across all resources used along the patient’s care pathway.
8	Cost-data analysis^e^	- Patients’ resource consumption and resulting costs based on care pathways and OSCI classification; - Descriptive analysis and multivariable models.

LoS length of stay, *LoT* length of time

^a^patient’s medical records and interviews of health care professionals were reviewed; ^b^costs included energy, equipment depreciation, general materials, and third-party contracts. The allocation formula considered the equipment used by COVID-19 patients, the area and the direct expenditures. 12-month average cost distribution was considered; ^c^LoT of nurse, nurse technician and physician on duty was assumed to be equally distributed between patient beds during each shift (shift’s length of time multiplied by the number of professionals working at each shift divided by the number of beds in the unit); ^d^price: excluding profit margins, and considering a 12-month average; ^e^results reported in international dollars (Int$)

This method begins by identifying the main goal of the analysis and by drawing the care process map (steps 1 and 2). Supplementary Fig. 1 presents the full care process made available to COVID-19 patients, presenting hospital units (emergency, general ward and intensive care, surgery room), main intensive care therapy strategies (pone position, renal replacement, plasma transfusion, ECMO and mechanical ventilation) and health professionals associated to patient treatment. Based on the review of patient’s resource use and stay at hospital units over their treatment, five different care pathways were identified: (i) Emergency + Ward, (ii) Emergency + Ward + ICU, (iii) Ward + ICU, (iv) ICU Only and (v) Emergency + ICU. Next, the method identifies the resources consumed at each stage of the patient’s care process (Step 3).

This information is then used to estimate the total cost of each resource group and department and to calculate the CCRs (Steps 4 and 5). The labor CCR was estimated from the hired work hours, dividing the total salary by the total contracted hours of each professional category. In cases in which there were no means to measure care activities length of time (i.e. no self-reported LoT was added to patients’ medical chart; no feasible chronoanalysis by an observer at the hospital unit was possible), health professionals availability per shift was assumed to be equally distributed between beds assigned to the treatment of COVID-19 patients in the respective hospital unit. For example, the nurse technician time consumption per patient in the ICU was calculated by multiplying the total number of professionals working per shift by the length in hours of each shift (no idle time considered) and then dividing it by COVID-19 patients’ beds in the ICU. The infrastructure CCR was estimated from the hospital units’ total monthly expenditures divided by the number of hours a unit is open per month and its number of beds. We calculated the practical capacity for each unit by considering the rooms or beds available and their monthly open availability. For example, for ward (inpatient) units, the number of beds available was multiplied by 24 hours a day and 30 days a month, while for surgical rooms, the rooms are usually available for 12 hours a day from Monday through Friday, and during the weekend, a reduced number of rooms is available. These real aspects of hospital routine were strictly followed for the capacity estimates.

Next, time estimates of resource use are acquired (Step 6). The average LoT necessary to perform care activities in the care cycle was reported by health professionals via online survey. A total of 42 physicians (multiple specialties), 7 physiotherapists, 2 pharmacists, and 6 psychologists submitted their self-reported care activities LoT. Patient’s LoS at each hospital unit was collected manually from patient’s EMRs.

Finally, total hospitalization cost per patient is calculated as the summed costs across all resources used along the patient’s care pathway, followed by cost-data analysis (Steps 7 and 8). The analysis investigated variability of care costs considering the patients’ care pathways followed during hospitalization and their scores on the OSCI scale. The overall median (interquartile range, IQR) cost per patient was calculated and descriptively presented per care pathway, allowing for the identification of those who incurred higher costs. Cost data were collected and analyzed in Brazilian currency, and results reported in international dollars (I$) as the mean (SD, standard deviation) and/or median (IQR, interquartile range). International dollars were calculated based on the purchasing power parity (PPP) value 2020 by conversion rate of I$1 = 0.44 Brazilian Reais (R$) [17].

### Statistical analyses

Multivariable models were created to evaluate the effect of care pathways and OSCI on overall patients’ treatment cost, estimating the mean cost values. As cost data presented high skewness, a log-link gamma generalized linear model (GLM) was applied, similarly to previous cost studies [18–21]. Log-link gaussian GLM was tested, but model assumptions did not hold. Log transformation of cost values was not used as mean cost estimates in the original scale could not be obtained.

Model diagnostic was done by using simulated residuals with the package ‘DHARMA’ in R. It was performed 1000 simulations to estimate the underlying distribution of the residuals. Then we assessed the fitting of the residuals to the underlying distribution through a QQ-plot and assessed heteroscedasticity with the graph of predicted vs. residuals and Levene test for the homogeneity of variance.

The main and interaction effects in all models were evaluated using the Wald chi-square test. Bonferroni post hoc test was used for pairwise comparisons. The presented effects were back transformed and reported as estimated means with 95% confidence intervals (CIs). The comparison of patient characteristics and costs by care pathway used Kruskal–Wallis and Dunn’s post-hoc tests for continuous variables and chi-square tests for categorical variables. For multiple pairwise comparisons, the *p* value was adjusted with the Benjamini–Hochberg method. A significance level of 0.05 was chosen for all analyses. Data collection was consolidated using Microsoft Excel and analyzed with R version 4.0.3 at RStudio version 1.4.1103.

## Results

### Patient characteristics

A sample of 208 patients was included in the study, which accounted for 17% of the total number of patients admitted at Hospital A and treated for COVID-19, regardless of the reason which led to hospital admission (i.e. emergency surgery, COVID-19, pregnancy, chronic disease treatment), through the emergency or hospital transference, from March 1st of 2020 to August 31st of 2020 [2]. Tables 2 and 3 describe patient’s characteristics and clinical evolution.

**Table 2:** Patient’s characteristics and clinical evolution

	Female 110 (53)	Male 98 (47)	Total 208
Age, median (IQR)	59 (46; 69)	58 (46; 68)	60 (46; 68)
18-44 years	23 (52)	21 (48)	44 (21)
45-64 years	49 (54)	42 (46)	91 (44)
65-84 years	33 (52)	31 (48)	64 (31)
> 85 years	4 (50)	4 (50)	8 (4)
Secondary diagnosis	83 (53)	74 (47)	157 (75)
Hypertension	62 (54)	53 (46)	115 (55)
Obesity	27 (59)	19 (41)	46 (22)
Diabetes	36 (49)	37 (51)	73 (35)
OSCI			
Dead	20 (47)	23 (53)	43 (21)
Severe	28 (53)	29 (47)	57 (27)
Mild	62 (57)	46 (43)	108 (52)
LoS (days), median (IQR)	8 (4; 16)	9 (3; 21)	8 (4; 17)

Data expressed as n (%), unless specified otherwise

*OSCI* Ordinal Scale for Clinical Improvement

**Table 3:** ICU patient’s characteristics and clinical evolution

	Female 41 (53)	Male 49 (47)	Total 90 (100)
Patient characteristics			
Age, median (IQR)	59 (46; 69)	59 (44; 71)	59 (45; 70)
Clinical evolution			
LoS (days), median (IQR)	16 (9; 22)	19 (12; 33)	17 (10; 30)
Mechanical ventilation	32 (48)	34 (52)	66 (73)
Deceased	13 (39)	20 (61)	33 (37)

Data expressed as n (%), unless specified otherwise

Considering the five care pathways identified, most patients (*n* = 118, 57%) followed the Emergency + Ward pathway (Table 4). The OSCI score of each case varied by care pathway (*p* <  0.001). Patients that visited the ICU (*n* = 90, 43%), classified as severe OSCI cases, followed in their majority (53%) the Emergency + Ward + ICU pathway or the Ward + ICU pathway(44%**)**. Deaths occurred in all pathways, but 33 (77% of total deaths) were observed on care pathways that included the ICU.

**Table 4:** Patient’s characteristics by care pathway

Variable	Emergency + Ward	Emergency + Ward + ICU	Ward + ICU	Emergency + ICU	ICU Only	p
	*N* = 118 (57)	*N* = 43 (21)	*N* = 27 (13)	*N* = 8 (4)	*N* = 12 (6)	
Age, median (IQR)	59 (46; 66)	61 (46; 70)	53 (42; 69)	64 (54; 77)	64 (51; 71)	0.479
Male gender	49 (42)	21 (49) ^a^	15 (56) ^a^	7 (88) ^a^	6 (50) ^a^	0.108
Secondary diagnosis	88 (56)	32 (20)	20 (13)	7 (4)	10 (6)	0.889
Hypertension	68 (58)	23 (54)	14 (52)	4 (50)	5 (42)	0.847
Obesity	26 (22)	10 (23)	7 (26)	1 (13)	2 (17)	0.925
Diabetes	42 (36)	12 (28)	10 (37)	4 (50)	4 (33)	0.861
OSCI						< 0.001
Dead	10 (9)	12 (28) ^a^	2 (7) ^a^	7 (88) ^a^	11 (92) ^a^	
Severe**	0 (0)	31 (72)	25 (93)	1 (13)	1 (8)	
Mild	108 (92)	0 (0)	0 (0)	0 (0)	0 (0)	
LoS, median (IQR)	5 (2; 9)	19 (13; 32)^a^	14 (9; 23)^a^	16 (7; 26)^a^	18 (8; 31)^b^	< 0.001

Data expressed as n (%), unless specified otherwise. Length of Stay (LoS) expressed as days

*OSCI* Ordinal Scale for Clinical Improvement, *LoS* Length of Stay

*p-value for the comparison between care pathways using Kruskal-Wallis or Chi-square test. **All patients admitted to the ICU were categorized as severe at OSCI. Pairwise comparison with Dunn’s test result: a. *p* < 0.001 vs. Emergency + Ward. b. *p* = 0.02 vs. Emergency + Ward

LoS analysis identified ICU care pathways as contributors to longer patient’s stays (*p* <  0.001) when compared to Emergency + Ward pathway, which presented a median LoS of 5 days (IQR 2; 9). LoS varied from 14 (IQR 9; 23) days to 19 (IQR 13; 32) days on pathways including the ICU. From a hospital unit perspective, the emergency unit presented a median LoS of 4 (IQR 3; 8) hours, the ward unit had a median LoS of 5 (IQR 2; 9) days and the ICU a 9 (IQR 5; 20) days median LoS.

### Cost analytics per care pathway and OSCI classification

**Fig. 1:**
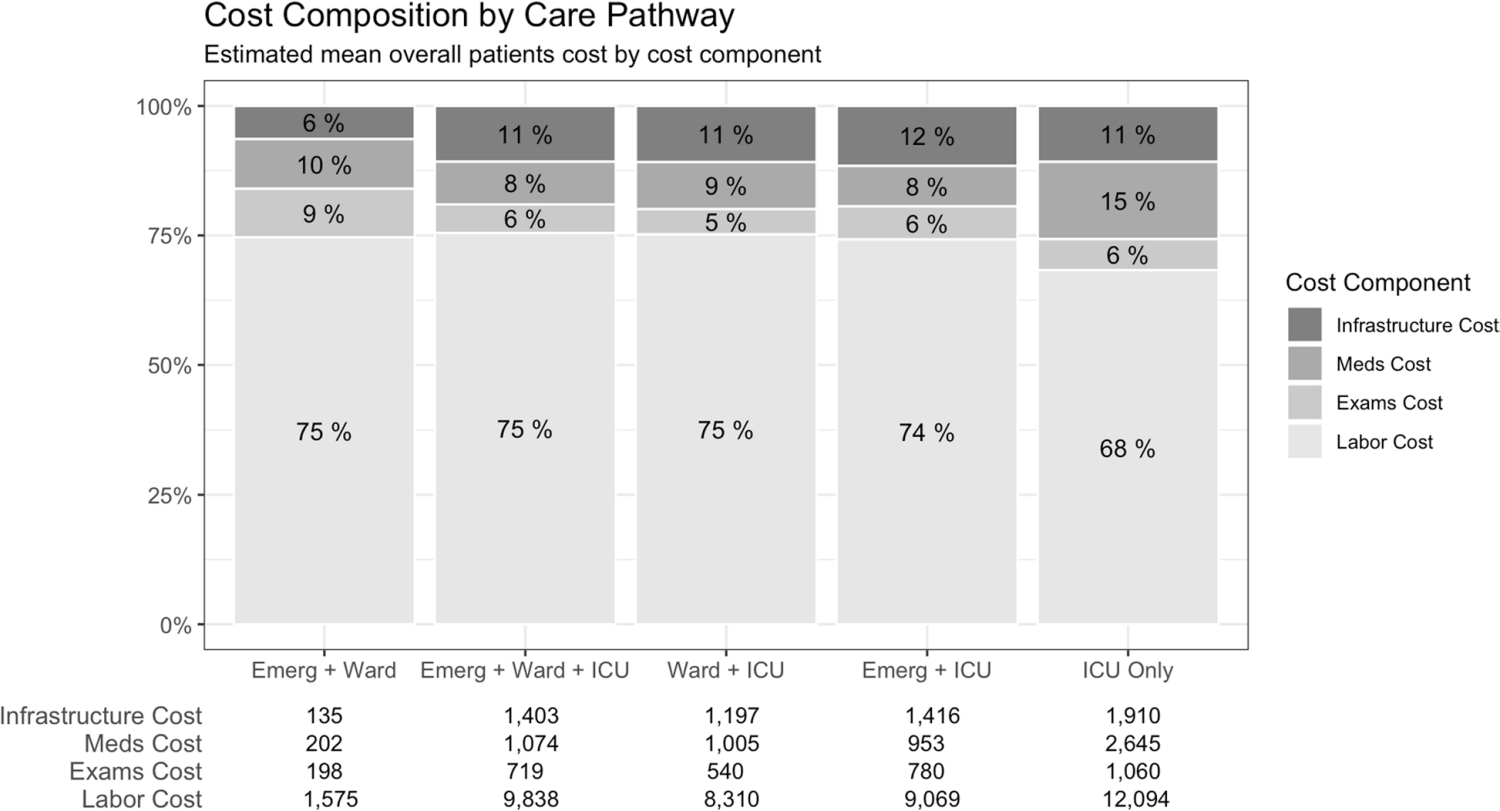
Composition of patients’ mean overall costs by cost component. Note: data expressed as mean and converted according to purchasing power parity (PPP) 2020

The median and mean overall costs per patient were, respectively, I$2879 (IQR 1215; 8140 and I$6818 (SD 9043) and daily costs per patient were I$410 (322; 575) and I$ 458 (164). Overall cost ranged across care pathways from I$1533 (IQR 813; 2649) in the Emergency + Ward pathway to I$13,519 (IQR 5637; 23,373) in the ICU Only pathway (Table 5). Labor cost component was responsible for 74% of mean overall treatment expenses, ranging from 68 to 75% between care pathways. Exams cost accounted for 6% (range 5 to 9%) of the overall cost (Fig. 1). Patients who received a mild disease classification on OSCI scale registered a median overall treatment cost of I$1444 (IQR 723; 2564). Although higher than mild disease cases, median cost of patients with severe disease score, I$7276 (IQR 4459; 14,139), and who died (highest score at OSCI), I$9534 (IQR 4863; 19,065), were similar between themselves.

**Table 5:** Treatment cost per patient by cost component and care pathway

Variables	Emerg. + Ward	Emerg. + Ward + ICU	Ward + ICU	Emerg. + ICU	ICU Only	Total
	*N* = 118 (57)	*N* = 43 (21)	*N* = 27 (13)	*N* = 8 (4)	*N* = 12 (6)	*N* = 208 (100)
Cost components						
Infrastructure	135 (136)	1403 (1161)^a^	1197 (1080)^a^	1416 (989)^a^	1910 (1532)^a^	687 (1005)
	98 (41; 177)	998 (516; 1798).	763 (511; 1654).	1361 (606; 2221).	1490 (706; 2652).	202 (76; 763)
Meds	202 (290)	1074 (1313)^a^	1005 (1198)^a^	953 (681)^a^	2646 (4452)^a^	656 (1432)
	90 (40; 225)	594 (270; 1398).	463 (249; 1497).	908 (570; 1351).	1189 (332; 2318).	225 (74; 613)
Exams	198 (192)	719 (589)^a^	540 (435)^a^	780 (807)^a^	1060 (1038)^a^	422 (521)
	155 (114; 222)	514 (296; 988).	362 (230; 762).	455 (399; 880).	752 (463; 1073).	225 (139; 440)
Labor	1575 (1540)	9838 (7479)^a^	8310 (7012)^a^	9069 (6448)^a^	12,094 (9711)^a^	5053 (6466)
	1157 (547; 1951)	7613 (4253; 12,073).	5672 (3328; 10,771).	8438 (3744; 14,224).	9368 (4322; 17,171).	2243 (891; 6029)
Total cost	2111 (2077)	13,033 (10,163)^a^	11,052 (9439)^a^	12,219 (8683)^a^	17,709 (16,020)^a^	6818 (9043)
	1533 (813; 2649)	10,388 (5450; 16,069).	7276 (4223; 14,512).	11,315 (5289; 18,605)	13,519 (5637; 23,373)	2879 (1215; 8140)
Daily cost	366 (116) 328 (302; 402)	534 (122)^a^ 536 (421; 643).	521 (93)^a^ 524 (451; 596).	739 (50)^abc^ 726 (704; 742)....	758 (87)^abc^ 757 (702; 813). ..	458 (164) 410 (322; 575)

Data expressed as mean cost (SD) and median cost (IQR) in International Dollar. Exchange rate 0.44 to Brazilian Reais. Multiple comparisons using Dunn’s test: a. *p* < 0.001 vs. Emergency + Ward, b. *p* < 0.001 vs. Emergency + Ward + ICU, c. *p* < 0.001 vs. Ward + ICU

#### Gamma regression models

The gamma regression multivariable analysis showed that both care pathways and OSCI, in each respective model, had a significant impact on overall treatment cost estimates. In both models, covariates (sex, age, and comorbidities) showed no significant effect on overall treatment costs and were removed from the final models. Residual analysis of both models showed a good fit to the underlying distribution and no problems regarding the homogeneity of variance (Suppl. Figs. 1 and 2).

**Fig. 2:**
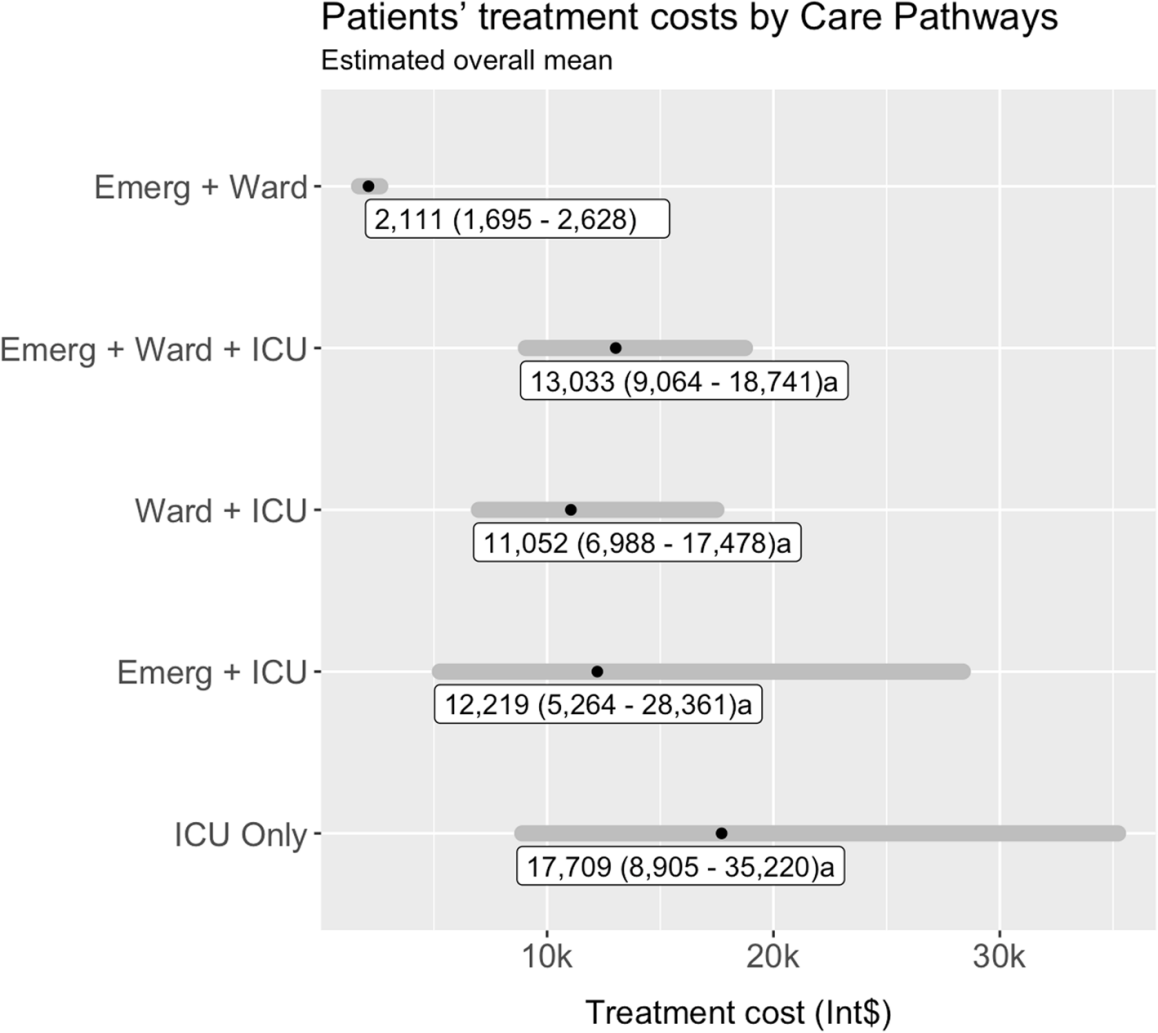
Estimated mean of overall patients’ treatment costs by care pathways. Note: data expressed as mean cost 95% CI (LL - UL) and converted according to purchasing power parity (PPP) 2020. Exchange rate 0.44 to Brazilian Reais. *P*-values for post-hoc pairwise comparison (Dunn’s Test with Benjamini–Hochberg adjustment): a, *p* < 0.001 vs. Emergency + Ward

In the final care pathway univariable model, pairwise comparison demonstrated that patients who followed ICU pathways, except for Emergency + ICU, had higher costs than those who followed Emergency + Ward pathway (*p* <  0.001). Estimated mean cost increase ranged from 424% (Ward + ICU) to 739% (ICU Only), as demonstrated by Eq. 1. Although patients who followed the Emergency + ICU pathway had 479% higher costs, pairwise comparison was not significant, which may be due to the small number of patients (*n* = 8) (*p* = 0.06). Overall cost differences between ICU pathways were not significant. Estimated overall costs by care pathway are presented in Fig. 2.

1 $$cCP={\exp}\left(7.65+1.82\ast EWI+1.66\ast WI+1.76\ast EI+2.13\ast I\right)$$

where:

cCP = mean total cost per care pathway,

Intercept refers to Emergency + Ward,

EWI = Emergency + Ward + ICU,

WI = Ward + ICU,

EI = Emergency + ICU,

I = ICU Only.

**Fig. 3:**
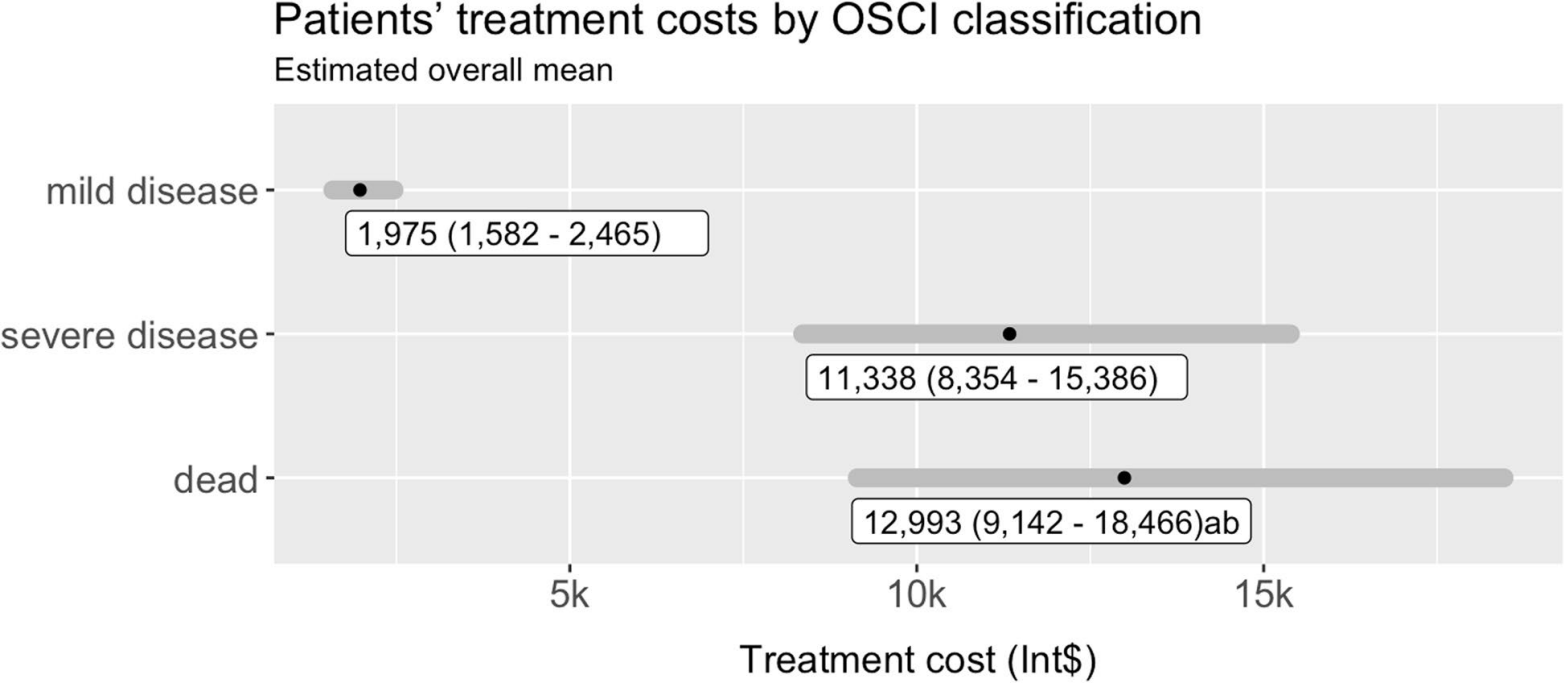
Estimated mean of overall patients’ treatment costs by OSCI classification. Note: data expressed as mean cost 95% CI (LL - UL) and converted according to purchasing power parity (PPP) 2020. Exchange rate 0.44 to Brazilian Reais *P*-values for post-hoc pairwise comparison (Dunn’s Test with Benjamini–Hochberg adjustment): a, *p* < 0.001 vs. mild disease; b, *p* < 0.001 vs. severe disease

In the final univariable model for OSCI effect, pairwise comparison presented significant differences between mild disease score and both severe disease and dead classifications (*p* < 0.001). However, there was no significant difference between the two (*p* = 0.99). The model is described by Eq. 2. Estimated mean overall treatment costs by OSCI classification are presented in Fig. 3.

2 $$cOSCI={\exp}\left(7.59+1.75\ast S+1.88\ast D\right)$$

where:

cOSCI = mean total cost per OSCI classification,

Intercept refers to mild disease,

S = severe disease,

D = dead.

## Discussion

This study presented the hospital cost estimations of in-hospital treatment for patients with COVID-19 and the cost implications of high variability due to the different care pathways that were followed, and the patients OSCI categorization in a middle-income country. All patients who were treated in the ICU during their hospital stay registered higher costs when compared with the Emergency + Ward pathway, while those admitted directly to the ICU had the highest overall costs. Independently of care pathway, labor costs were the dominant cost component (74%), followed by infrastructure (10%), medication (10%) and exams (6%). Our study encountered a median overall cost per patient of I$2879 (IQR 1215; 8140) and a median daily cost of I$410 (IQR 322; 575).

A leading impact of labor expenses was also reported by other studies that applied the TDABC method. APBdS Etges et al. [22] analyzed cholecystectomy procedure and their findings showed labor to account for ~ 68% of hospital costs. Y Anzai et al. [23] analyzed costs related to abdomen and pelvis computed tomography exam and their results demonstrated that labor was responsible for 80% of the direct costs to the academic medical center. In a study done by M Schuster and T Standl [24], 53% of anesthesia procedures costs were related to personnel. Impact of cost components, such as labor, on overall cost may vary according to the selected cost components in the analysis. Data granularity, regional characteristics (i.e health professionals’ salaries, medical supplies’ prices) and hospital financial structure may also contribute to this variability.

Median reimbursement by SUS of hospital costs related to COVID patients’ treatment was I$1496 (IQR 666; 8596) at the same hospital and period of our study [25]. In cases of multiple treatments per patient, this reimbursement gap might have been smaller. Nevertheless, this gap presents a major challenge for economic sustainably of health services and highlights the relevance of microcosting studies to better understand resource consumption and real cost of treatments.

To date, few microcosting studies on patients with COVID-19 have been reported. In our analysis, medication costs represented ~ 10% of overall costs, but a previous study reported these costs, including medical supplies, as the second highest cost, comprising 28% of the mean costs [26]. M Ghaffari Darab et al. [26] estimated the overall economic burden based on a single-center cohort from Iran. They collected cost information from 477 patients treated for COVID-19 at a referral university hospital in Fars Province from March to July 2020. They found a mean cost per patient of $3755 (SD 4684), with 41% of the cost being related to intensive and general care and 28% related to medications and medical supplies. They estimated a mean indirect cost related to premature death and economic production loss of I$11,634. I Edoka et al. [27] conducted a microcosting analysis of COVID-19 inpatient care from a public health perspective in South Africa. Their study combined local cost inputs with parameters for diagnosis and treatment and referenced daily resource consumption obtained from the literature. Regarding levels of care, general wards, high care wards and ICUs were considered [27]. The average daily cost, in 2020, varied by level of care, from ~USD$119 to ~USD$278 depending on oxygen supplementation in general wards, ~USD$278 in high care wards, and ~ USD$798 to ~USD$829, depending on respiratory support in the ICU. In Brazil, A Miethke-Morais et al. [28] performed a micro and macro-costing analysis of admissions from all consecutive patients admitted from March 30 to June 30, 2020, with suspected, probable, or confirmed COVID-19 (77%) at a quaternary hospital located in the city of São Paulo. Drugs, laboratory tests, radiologic exams, blood components and nutrition requirements consumed by each patient and, hospital supplies, human resources, and hospital fixed costs by bed-day per unit were identified and quantified. Cost analysis did not follow TDABC method as patients’ LoS and LoT of health professionals’ care activities were not taken into consideration. The average cost per admission (I$30,582), and the overall daily cost (I$2224) were calculated considering the total sum of costs and LoS. Age strata > 69 years, COVID-19, comorbidities, use of mechanical ventilation or dialysis, surgery and outcomes were associated with higher costs.

Our study encountered a mean overall cost per patient of I$6818 (SD 9043), and although we did not include hospital supply costs, cost per patient was ~ 1.8 times higher than those by M Ghaffari Darab et al. [26]. A Miethke-Morais et al. [28] overall results were ~ 4.5 times higher than ours. In contrast to our study, their analysis included costs related to medical supply, nutrition, personal protective equipment and nonmedical staff. Nonmedical staff overhead alone accounted for an average of 53% hospital units’ direct costs. Mean daily results were calculated as the sum of all patients’ cost divided by the sum of all patients’ LoS. Our study decided for a more precise approach as daily costs were calculated by dividing each patient’s total cost by their own total LoS. We applied the TDABC method, a well-suited approach to understanding the complexity of costs in health care [8], as it computes patient-level cost information considering which resources are needed and details how, where and for how long resources are used within an episode of care [3–6].

Our analysis showed that the patients’ care pathway, as well as their OSCI classification, influences hospitalization costs. Patients admitted directly to the ICU and care pathways that included the ICU unit incurred higher overall costs, as did patients classified as having severe COVID-19 and those who died. In the United States, a previous study revealed that patients who received treatment using invasive mechanical ventilation in the ICU unit incurred costs nearly six times greater than those patients who did not require intensive care [10]. A similar analysis performed with data from hospitalized patients in Iran encountered a difference of four times greater [26]. In our study, this difference was nearly six times greater. In Brazil, nearly 40% of hospitalized patients with COVID-19 needed care in the ICU unit, and 32.5% required invasive mechanical ventilation [29]. Health decision makers should promptly act to ensure that structural and labor resources are readily available in future health care crises, avoiding resource shortages and, as result, potential higher labor costs and decrease on quality of care.

The TDABC method was recently used in an Italian research [30] to achieved greater granularity of cost information per care pathway. Comparison between in-hospital care pathways presented a ~ 3 times increase in daily cost between the lowest (I$730) and the highest complexity (I$2156) care pathways. In our study, the highest complexity care pathway (I$757) was ~ 2 times more expensive than the care pathway with the least complexity (I$328).

These differences of daily cost per care pathway demonstrates the potential benefits of considering a risk adjustment coefficient to reimbursement strategies. The value of such decisions highlights the potential impact of microcosting studies in the improvement of decision-making processes when defining general reimbursement strategies for healthcare systems.

Our study contributes to bridging an existing information gap in this field. By understanding the differences in costs explained by the patients’ clinical status and care pathways, the process of designing and defining reimbursement strategies for COVID-19 can increase its accuracy. OSCI and the care pathway followed by a patient are two data points that can be recorded as part of the hospital routine and be made available to provider and payor managers as a driver for reimbursement parameters. Added to the cost information, the consideration of the patients’ clinical conditions and, if possible, outcomes should be done to establish a sustainable health policy for COVID-19 reimbursement in a middle-income country such as Brazil.

### Limitations and future studies

Medical supply costs were not included in the estimated COVID-19 patient costs due to limitations on available hospital data and overhead costs, such as administrative and management costs, as they were not allocated down to the patient level. Self-report length of time of health care activities was used as there were no means to measure LoT. Although this method is known to be less precise than an actual measurement [31], health professionals were performing these activities daily for several months at the time they were invited to answer the survey. Thus, they had both experienced the impact of the pandemic in their daily activities as well as a fresh account of LoT, reducing potential discrepancies [32]. It was not possible to collect data from all patients who met the inclusion criteria in the period of analysis due to a shortage of research staff. However, to reduce selection bias, we used aleatory sampling. This analysis comprises a sample of patients from a single hospital in southern Brazil, with a small sample per care pathway and thus with a small generalizability, and not being a focus to compare COVID-19 with non-COVID-19 patients in terms of costs. It was not possible to create a model considering both treatment and care pathways due to the small number of patients who received therapeutic strategies in each care pathway; thus, the connection between the care pathway and treatments needs further investigation. The period of data collection corresponds to the first wave of COVID-19 in Brazil, and treatment practices changed during and since the period of analysis.

The expansion of the data sample, including multiple hospitals, is strongly recommended to achieve more representative cost information for COVID-19 patient treatment. In addition, the inclusion of variables such as patients’ clinical condition upon arrival and main outcomes in multivariable models is recommended to enrich the discussion of value-reimbursement parameters for COVID-19.

## Conclusion

This was the first microcosting study of COVID-19 treatment costs that applied TDABC and, by exploring the power of this method, demonstrated how patient-level costs vary as a function of patients’ care pathway and their OSCI classification. The evaluation of the variability of patients’ complexity and profiles of hospital resource consumption provides valuable information to drive the design of future reimbursement policies that ensure the financial sustainability and quality of health care services.

The presented results and discussions about the use of accurate cost information to propose value reimbursement strategies that can contribute to sustainable health policies in middle-income countries such as Brazil showed that the application of the TDABC method allows for a better understanding of cost compositions and drivers of costs.

## Supplementary Information

**Additional file 1: Suppl. Fig. 1.** The COVID-19 care pathways and specific resources, describing macro activities, number of cases, and mean patients LoS spent at each macro activity.

**Additional file 2: Suppl. Fig. 2.** Residual analysis - QQ plot and predicted vs residual plots.

**Additional file 3: Suppl. Fig. 3.** Residual analysis - QQ plot and predicted vs residual plots.

## Data Availability

The data that support the findings of this study are available on request from the corresponding author. The data are not publicly available due to privacy or ethical restrictions.

## Abbreviations

TDABC: Time-driven activity-based costing
ICU: Intensive Care Unit
RT–PCR: Reverse Transcription-polymerase Chain Reaction
OSCI: Ordinal Scale for Clinical Improvement
ECMO: Extracorporeal Membrane Oxygenation
LoS: Hospital Length of Stay
EMRs: Electronic Medical Records
CCR: Capacity Cost Rate
LoT: Length of Time
IQR: Interquartile Range
PPP: Purchasing Power Parity
CIs: Confidence Intervals

## References

[1] Nicola M, Alsafi Z, Sohrabi C, Kerwan A, Al-Jabir A, Iosifidis C, Agha M, Agha R (2020). The socio-economic implications of the coronavirus pandemic (COVID-19): a review. Int J Surg.

[2] Etges APBS, Bertoglio Cardoso R, Marcolino MS, Brasil Ruschel K, Coutinho AP, Pereira EC, Anschau F, Aranha F, Carrilho F, Vietta G (2021). The economic impact of COVID-19 treatment at a hospital-level: investment and financial registers of Brazilian hospitals. J Health Econ Outcomes Res.

[3] Kaplan R, Witkowski M, Abbott M, Guzman A, Higgins L, Meara J, Padden E, Shah A, Waters P, Weidemeier M (2014). Using time-driven activity-based costing to identify value improvement opportunities in healthcare. J Healthcare Manag Am Coll Healthcare Executives.

[4] Keel G, Savage C, Rafiq M, Mazzocato P (2017). Time-driven activity-based costing in health care: a systematic review of the literature. Health Policy.

[5] Allin O, Urman RD, Edwards AF, Blitz JD, Pfeifer KJ, Feeley TW, Bader AM (2019). Using time-driven activity-based costing to demonstrate value in perioperative care: recommendations and review from the Society for Perioperative Assessment and Quality Improvement (SPAQI). J Med Syst.

[6] Etges APBS, Ruschel KB, Polanczyk CA, Urman RD (2020). Advances in value-based healthcare by the application of time-driven activity-based costing for inpatient management: a systematic review. Value Health.

[7] da Silva Etges APB, Cruz LN, Notti RK, Neyeloff JL, Schlatter RP, Astigarraga CC, Falavigna M, Polanczyk CA (2019). An 8-step framework for implementing time-driven activity-based costing in healthcare studies. Eur J Health Econ.

[8] Kaplan RS, Porter ME (2011). How to solve the cost crisis in health care. Harv Bus Rev.

[9] Lee T, Porter M. The strategy that will fix healthcare. In: Harvard Business Review. Boston: Harvard Business School; 2013.

[10] Di Fusco M, Shea KM, Lin J, Nguyen JL, Angulo FJ, Benigno M, Malhotra D, Emir B, Sung AH, Hammond JL (2021). Health outcomes and economic burden of hospitalized COVID-19 patients in the United States. J Med Econ.

[11] McBain RK, Jerome G, Warsh J, Browning M, Mistry B, Faure PAI, Pierre C, Fang AP, Mugunga JC, Rhatigan J (2016). Rethinking the cost of healthcare in low-resource settings: the value of time-driven activity-based costing. BMJ Glob Health.

[12] Brazil. Ministério da Saúde (MS): SIGTAP - Sistema de Gerenciamento da Tabela de Procedimentos, Medicamentos e OPM do SUS In*.* Brasília: Ministério da Saúde (MS); 2022.

[13] Saúde Md: Diretriz Metodológica: Estudos de Microcusteio Aplicados a Avaliação Econômica em Saúde. In*.*: Ministério da Saúde Brasília, Brasil; 2020.

[14] Hollander JE, Sites FD. The transition from reimagining to recreating health care is now. NEJM Catalyst Innovations in Care Delivery. 2020;1(2).

[15] Transparência COVID-19 - Painel Saúde [https://infografico-covid.procempa.com.br/].

[16] Blueprint W (2020). Novel coronavirus COVID-19 therapeutic trial synopsis.

[17] Brazil PPP (2020). Conversion rate, LCU per international Dollar.

[18] Etges APBdS, Marcolino MAZ, Ogliari LA, de Souza AC, Zanotto BS, Ruschel R, et al. Moving the Brazilian ischaemic stroke pathway to a value-based care: introduction of a risk-adjusted cost estimate model for stroke treatment. Health Policy Plan. 2022;37(9):1098–106. 10.1093/heapol/czac058 35866723

[19] Gong J, Wang Y, Huang ST, Chiu HC (2022). Study of hospitalization costs in patients with cerebral ischemia based on E-CHAID algorithm. J Healthc Eng.

[20] Gregori D, Petrinco M, Bo S, Desideri A, Merletti F, Pagano E (2011). Regression models for analyzing costs and their determinants in health care: an introductory review. Int J Qual Health Care.

[21] Dodd S, Bassi A, Bodger K, Williamson P (2006). A comparison of multivariable regression models to analyse cost data. J Eval Clin Pract.

[22] Etges APBdS, Cruz LN, Schlatter RP, Neyeloff J, Ferranti E, Kopittke L, et al. Identifying cost-saving opportunities for surgical care via multicenter time-driven activitybased costing (TDABC) analysis as exemplarily shown for cholecystectomy. J Hospital Manag Health Policy. 2021;6. 10.21037/jhmhp-21-34.

[23] Anzai Y, Heilbrun ME, Haas D, Boi L, Moshre K, Minoshima S, Kaplan R, Lee VS (2017). Dissecting costs of CT study: application of TDABC (time-driven activity-based costing) in a tertiary academic center. Acad Radiol.

[24] Schuster M, Standl T (2006). Cost drivers in anesthesia: manpower, technique and other factors. Curr Opin Anesthesiol.

[25] TabNet. https://datasus.saude.gov.br/informacoes-de-saude-tabnet/. Accessed 10 Nov 2022.

[26] Ghaffari Darab M, Keshavarz K, Sadeghi E, Shahmohamadi J, Kavosi Z (2021). The economic burden of coronavirus disease 2019 (COVID-19): evidence from Iran. BMC Health Serv Res.

[27] Edoka I, Fraser H, Jamieson L, Meyer-Rath G, Mdewa W. Inpatient care costs of COVID-19 in South Africa’s public healthcare system. Int J Health Policy Manag. 2021;11(8):1354–61. 10.34172/ijhpm.2021.24 PMC9808349 33949817

[28] Miethke-Morais A, Cassenote A, Piva H, Tokunaga E, Cobello V, Rodrigues Gonçalves FA, Dos Santos LR, Trindade E, Carneiro DALA, Haddad L (2021). COVID-19-related hospital cost-outcome analysis: the impact of clinical and demographic factors. Braz J Infect Dis.

[29] Marcolino MS, Ziegelmann PK, Souza-Silva MVR, Nascimento IJB, Oliveira LM, Monteiro LS, Sales TLS, Ruschel KB, Martins KPMP, Etges APBS (2021). Clinical characteristics and outcomes of patients hospitalized with COVID-19 in Brazil: results from the Brazilian COVID-19 registry. Int J Infect Dis.

[30] Foglia E, Ferrario L, Schettini F, Pagani MB, Dalla Bona M, Porazzi E: COVID-19 and hospital management costs: the Italian experience. In: BMC Health Serv Res*.* vol. 22, 2022/08/04 edn; 2022: 991. 10.1186/s12913-022-08365-9 PMC9351199 35922849

[31] Short ME, Goetzel RZ, Pei X, Tabrizi MJ, Ozminkowski RJ, Gibson TB, Dejoy DM, Wilson MG (2009). How accurate are self-reports? Analysis of self-reported health care utilization and absence when compared with administrative data. J Occup Environ Med.

[32] Kjellsson G, Clarke P, Gerdtham UG (2014). Forgetting to remember or remembering to forget: a study of the recall period length in health care survey questions. J Health Econ.

