# Supplementary figures and images for: Comparison of COVID-19 hospitalization costs across care pathways: a patient-level time-driven activity-based costing analysis in a Brazilian hospital
Source: BMC Health Serv Res. 2023 Feb 24;23:198. doi: 10.1186/s12913-023-09049-8 (PMC9955521; doi:10.1186/s12913-023-09049-8)

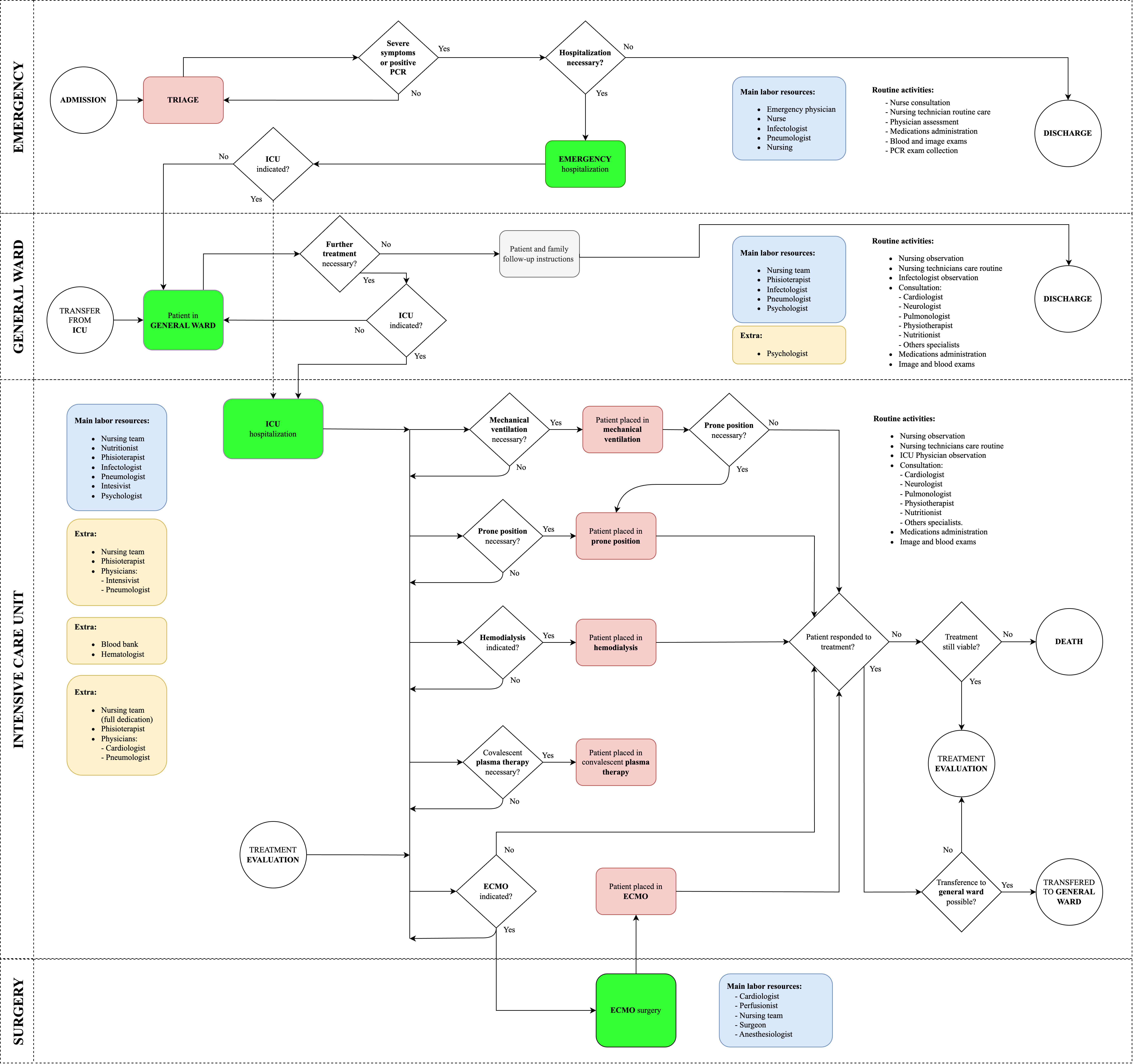

Supplement: Supplementary file 1 — Additional file 1: Suppl. Fig. 1. The COVID-19 care pathways and specific resources, describing macro activities, number of cases, and mean patients LoS spent at each macro activity. [file 12913_2023_9049_MOESM1_ESM.png]

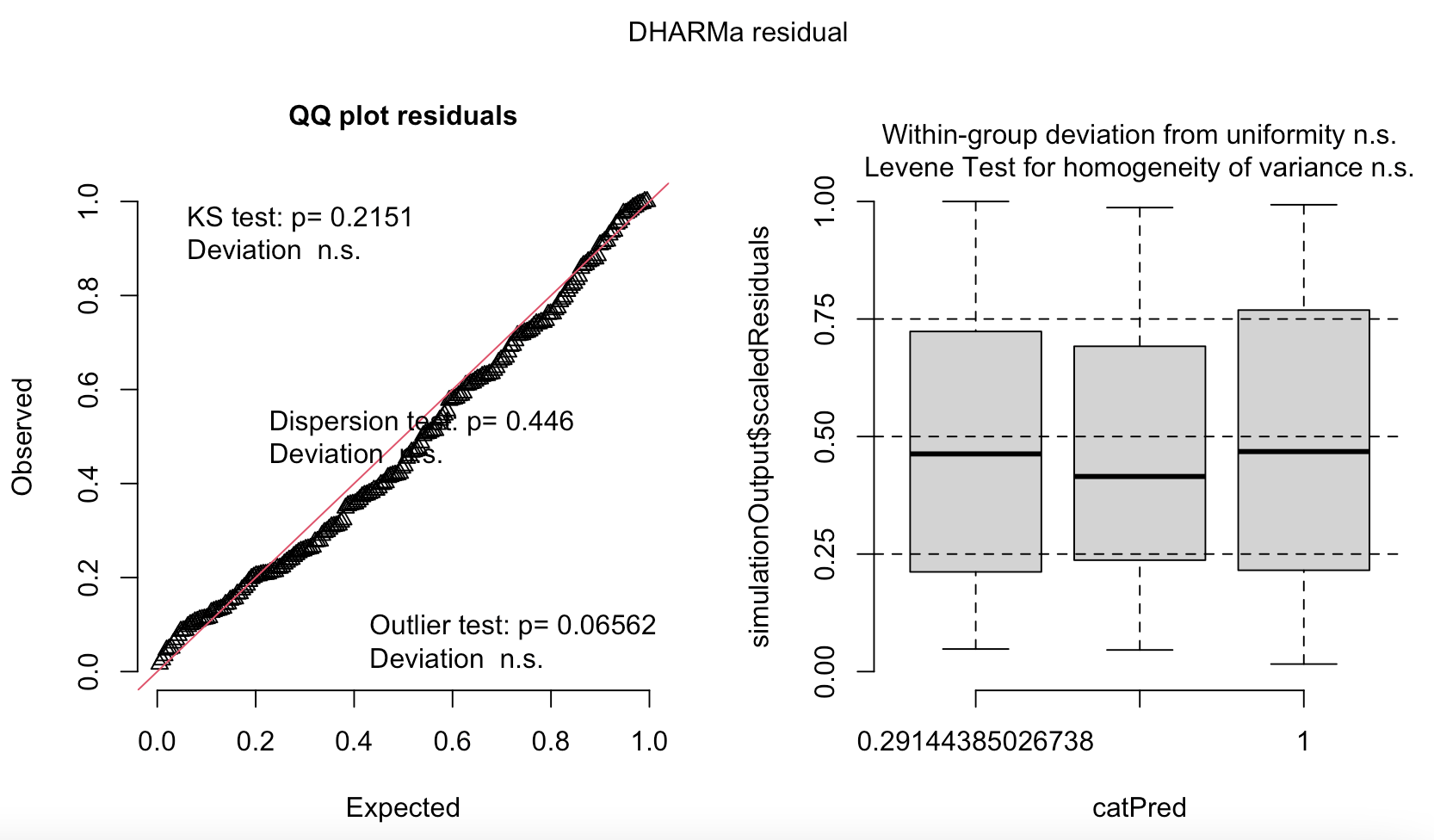

Supplement: Supplementary file 2 — Additional file 2: Suppl. Fig. 2. Residual analysis - QQ plot and predicted vs residual plots. [file 12913_2023_9049_MOESM2_ESM.png]

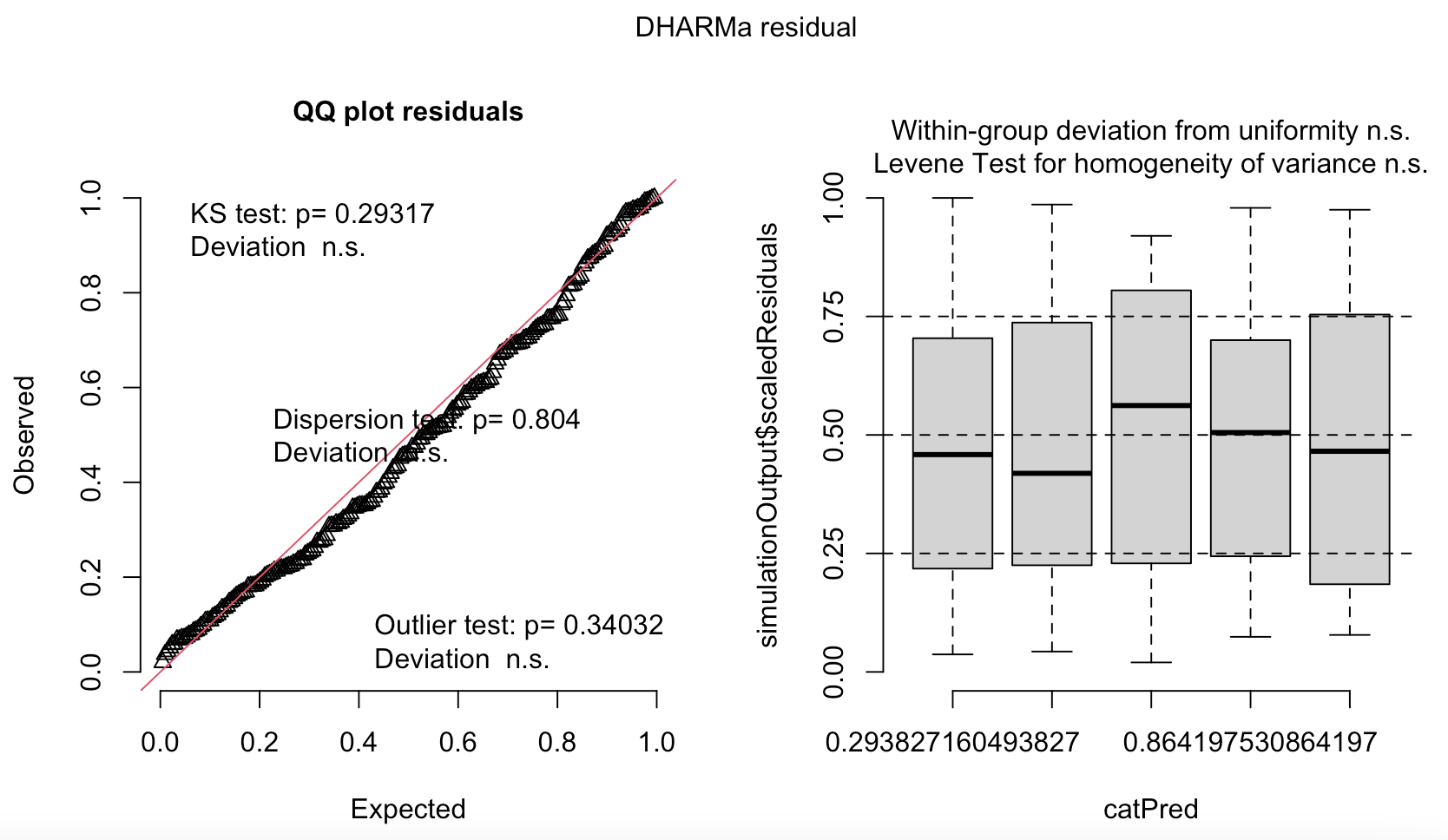

Supplement: Supplementary file 3 — Additional file 3: Suppl. Fig. 3. Residual analysis - QQ plot and predicted vs residual plots. [file 12913_2023_9049_MOESM3_ESM.png]
